# Critical Comparison of MaxCal and Other Stochastic Modeling Approaches in Analysis of Gene Networks

**DOI:** 10.3390/e23030357

**Published:** 2021-03-17

**Authors:** Taylor Firman, Jonathan Huihui, Austin R. Clark, Kingshuk Ghosh

**Affiliations:** 1 Molecular and Cellular Biophysics, University of Denver, Denver, CO 80208, USA; taylor.firman@childrenscolorado.org (T.F.); Austin.Clark@du.edu (A.R.C.); 2 Department of Physics and Astronomy, University of Denver, Denver, CO 80208, USA; Jonathan.Huihui@du.edu

**Keywords:** gene network, inference, Maximum Caliber

## Abstract

Learning the underlying details of a gene network with feedback is critical in designing new synthetic circuits. Yet, quantitative characterization of these circuits remains limited. This is due to the fact that experiments can only measure partial information from which the details of the circuit must be inferred. One potentially useful avenue is to harness hidden information from single-cell stochastic gene expression time trajectories measured for long periods of time—recorded at frequent intervals—over multiple cells. This raises the feasibility vs. accuracy dilemma while deciding between different models of mining these stochastic trajectories. We demonstrate that inference based on the Maximum Caliber (MaxCal) principle is the method of choice by critically evaluating its computational efficiency and accuracy against two other typical modeling approaches: (i) a detailed model (DM) with explicit consideration of multiple molecules including protein-promoter interaction, and (ii) a coarse-grain model (CGM) using Hill type functions to model feedback. MaxCal provides a reasonably accurate model while being significantly more computationally efficient than DM and CGM. Furthermore, MaxCal requires minimal assumptions since it is a top-down approach and allows systematic model improvement by including constraints of higher order, in contrast to traditional bottom-up approaches that require more parameters or ad hoc assumptions. Thus, based on efficiency, accuracy, and ability to build minimal models, we propose MaxCal as a superior alternative to traditional approaches (DM, CGM) when inferring underlying details of gene circuits with feedback from limited data.

## 1. Introduction

A common goal in synthetic biology of single cells is to design and introduce small circuits to optimize certain behaviors [1,2,3,4,5,6,7,8,9,10]. However, this requires quantitative knowledge about the details of these circuits and their subsequent optimization. Circuit characteristics are often studied by single-cell measurements using fluorescent markers on few proteins [11]. These traditional measurements—with the exception of a few emerging technologies that simultaneously monitor transcription and translation [12,13]—cannot visualize many other underlying species that are involved in dictating gene expression dynamics. For example, nucleic acids, protein-complexes, protein-nucleic acid complexes remain invisible. So, how do we extract information from such limited data? One possible solution is to track the stochastic gene expression trajectories of the fluorescently tagged proteins and use the noisy time series data to learn about the underlying invisible network [14,15,16,17,18,19]. This data mining scheme requires building stochastic models that are typically bottom-up and can be divided in two major categories. The first approach builds detailed models (DM) based on a pre-specified reaction network that include tagged proteins and other molecules such as promoter, protein-promoter complexes, etc. [20,21]. These detailed reaction schemes are usually simulated using Gillespie algorithm [22,23] to generate noisy trajectories and analyze circuit properties. However, the same DMs can be used for inference as well. The detailed reactions—written as chemical master Equation (CME) [24] and solved using Finite State Projection method [25,26]—can yield probabilities of numbers of molecules as a function of rate parameters. Consequently, parameter inferences can be done by trying to match the measured noisy trajectory. The second approach builds coarse-grain models (CGM) using a smaller set of reactions where effective reaction rates are implemented by assuming an ad hoc mechanism that accounts for invisible species. CGMs can be made arbitrarily complex with additional fit parameters but we consider only the minimal CGMs due to their computational efficiency and an alternate to DM. Although CGMs are typically used to describe average protein numbers [27,28], CMEs can be constructed for CGMs [29,30,31,32] and solved via FSP to describe probability distributions. Both models (CGM and DM) have merits and demerits over each other. DM’s are by definition more detailed and are expected to be accurate but require invoking multiple molecular species that are not always visible. Consequently, DM’s involve multiple parameters and a much bigger phase space. Thus, accuracy comes at the expense of computational cost. Minimal CGM’s on the other hand minimize computational burden by using effective rates based on pre-specified mechanisms. As a result, these models can be less accurate but efficient. The accuracy vs. efficiency dilemma raises two critical questions: how costly is a DM and how inaccurate is a CGM? Are CGM’s accurate enough or do we need to build a third class of models that are still feasible and yet more accurate than CGM?

We have recently introduced the principle of Maximum Caliber (MaxCal) to model and infer underlying details of gene networks using stochastic gene expression data [32,33,34,35,36]. The adoption of MaxCal-based inference for small gene networks provides a third approach that resolves the accuracy vs. efficiency dilemma. Maximum Caliber (MaxCal) is analogous to the Maximum Entropy (MaxEnt) principle but applied to the distribution of paths/trajectories instead of states [37,38]. Similar to MaxEnt, MaxCal maximizes path entropy (caliber) subject to constraints. In the application to gene networks, MaxCal starts with a minimal set of constraints on proteins whose numbers are followed by fluorescent tags. This makes MaxCal directly amenable to model measured time series data of gene expression while not invoking additional mechanisms or auxiliary species [32]. Consequently, MaxCal avoids ad hoc assumptions on mechanisms inherent in CGM while bypassing the challenge of increased phase space inherent in DM. In addition, the top-down nature of MaxCal allows systematic model building via the imposition of constraints of a higher order on top of the minimal constraints it starts with—allowing the user to improve accuracy as needed. This is in contrast to bottom-up approaches of DM and CGM, which work on the premise that a given model must be pre-specified based on pre-imposed assumptions. Since each mechanism/model can be very different from each other, it is difficult to systematically add higher order terms in a given model to increase model complexity. Instead, a new model must be generated every time and the process of testing must start all over.

In this work we show how MaxCal provides a model that is minimal and more accurate than CGM while also being as efficient (or more) as CGM. We establish this with two basic genetic circuits: Single Gene Auto-activation (SGAA) [34] and Toggle Switch (TS) where two genes mutually repress each other [1,21,33]. We first generate synthetic data to be used as input by simulating the respective reaction networks with details of protein and nucleic acid dynamics. We subject three different models (DM, CGM and MaxCal) to infer parameters from this synthetic data for which actual model parameters are known. This serves as the benchmark for accuracy between the three models of DM, CGM, and MaxCal. By noting the computation time needed, we can assess accuracy and efficiency of all three models. The quantitive comparison shows that for small circuits used by synthetic biologists, MaxCal is efficient and reasonably accurate.

## 2. Materials and Methods

In this section, we describe the methods involved in generating synthetic data of stochastic gene expression using known parameters that serve as a benchmark to test the accuracy of different models. We also describe different methods of analysis to infer model parameters from this stochastic data.

### 2.1. Single Gene Auto-Activation (SGAA) Circuit

We first demonstrate MaxCal’s performance on a single gene auto activation circuit (SGAA). This circuit consists of a single protein that enhances its own production and has been studied extensively in different biological contexts [30,31,39,40,41,42,43,44].

#### 2.1.1. Generating Synthetic Data

We first describe the model introduced by Elston et al. [20] to generate synthetic data mimicking experiment.

(1) $$\begin{array}{ccc} & & \alpha \overset{g}{\to }\alpha +a\;\;;\;\;a\overset{d}{\to }⊘\\ & & a\overset{p}{\to }a+A\;\;;\;\;A\overset{r}{\to }⊘\\ & & A+A\begin{array}{c} \overset{f_{d}}{\to }\\ \overset{}{\underset{b_{d}}{\leftarrow }} \end{array}A_{2}\\ & & \alpha +A_{2}\begin{array}{c} \overset{f_{p}}{\to }\\ \overset{}{\underset{b_{p}}{\leftarrow }} \end{array}\alpha^{*}\;\;;\;\;\alpha^{*}\overset{g^{*}}{\to }\;\alpha^{*}+a \end{array}$$

where *a* is mRNA transcribed from gene α with rate *g*, and gets degraded with rate *d*. Protein *A* is translated from *a* with basal rate *p* and degraded with rate *r*. Two *A* molecules complex to form a dimer $A_{2}$ with rate $f_{d}$, and dissociate back to monomers with rate $b_{d}$. The dimer $A_{2}$ can bind and unbind to the promoter with rate $f_{p}$ and $b_{p}$, respectively. In the bound form, the promoter gets converted to the activated state $\alpha^{*}$ producing mRNA at an enhanced rate $g^{*}$ much greater than *g*. Thus, *A* promotes self-activation. Synthetic input trajectories were generated using a Gillespie algorithm to simulate Equation (1) with $d=0.2\;s^{-1}$, $p=0.02\;s^{-1}$, $f_{d}=5.0\times 10^{-3}\;s^{-1}$, $b_{d}=50.0\;s^{-1}$, $f_{p}=6.0\times 10^{-3}\;s^{-1}$, $b_{p}=3.0\times 10^{-5}\;s^{-1}$, $g=0.05\;s^{-1}$, $g^{*}=0.5\;s^{-1}$, $r=1.0\times 10^{-3}\;s^{-1}$. These parameters and the reaction scheme (1) were chosen for three primary reasons: First, dimers are typical stoichiometry for regulatory proteins binding to the promoter site [20]. Next, the parameters produce noisy switch like trajectory and were used earlier to test MaxCal’s inferential power [35]. Third, the rate parameter values and resulting switching times (few hours) are typical in realistic circuits [5]. Finally, to further mimic experiment, ten replicates of one hundred trajectories—each trajectory seven days long—were considered.

#### 2.1.2. The Detailed Model for Inference

Next, we describe the three models (DM, CGM, and MaxCal) used to infer the underlying details of the circuit from the synthetic data generated. We start with the DM model first. The detailed model (DM) of SGAA is built by considering the promoter in its basal state (α), activated state ($\alpha^{*}$), and the protein *A*. The reaction scheme is given by

(2) $$\begin{array}{ccc} & & \alpha \overset{g_{1}}{\to }\alpha +A\;\;;\;\;A\overset{r_{1}}{\to }⊘\\ & & \alpha +A\begin{array}{c} \overset{f_{1}}{\to }\\ \overset{}{\underset{b_{1}}{\leftarrow }} \end{array}\alpha^{*}\;\;;\;\;\alpha^{*}\overset{g_{1}^{*}}{\to }\;\alpha^{*}+A \end{array}$$

The effective protein production rate in the non-activated and activated forms are $g_{1}$ and $g_{1}^{*}$, respectively. Upon defining the reaction network by Equation (2), the system can be described by a chemical master Equation (CME) to compute probability $P(N_{A},t)$ of observing $N_{A}$ number of *A* proteins at time *t*. The CME is given by

(3) $$\begin{array}{ccc} \frac{dP(N_{A},\alpha ,t)}{dt} & = & \left(g_{1}\alpha +g_{1}^{*}\left(1-\alpha \right)\right)P\left(N_{A}-1,\alpha ,t\right)\\ & - & \left(g_{1}\alpha +g_{1}^{*}\left(1-\alpha \right)\right)P\left(N_{A},\alpha ,t\right)\\ & + & r_{1}\left(N_{A}+1\right)P\left(N_{A}+1,\alpha ,t\right)-r_{1}N_{A}P\left(N_{A},\alpha ,t\right)\\ & + & \left(N_{A}+1\right)f_{1}\left(1-\alpha \right)P\left(N_{A}+1,1-\alpha ,t\right)-N_{A}f_{1}\alpha P\left(N_{A},\alpha ,t\right)\\ & + & b_{1}\alpha P\left(N_{A}-1,1-\alpha ,t\right)-b_{1}\left(1-\alpha \right)P\left(N_{A},\alpha ,t\right) \end{array}$$

where, α=1 and 0 denote the basal and the activated state ($\alpha^{*}$) of the promoter, respectively. The CME can be expressed in the matrix form as

(4) $$\frac{dP_{i}\left(t\right)}{dt}=\sum_{ij}W_{ij}P_{j}\left(t\right)$$

where, states (*i*) are given by different values of $N_{A}$ and α and their time dependent probability is $P_{i}\left(t\right)$. The transition matrix *W* is a function of rate parameters $g_{1},r_{1},f_{1},b_{1},g_{1}^{*}$ and can be constructed from Equation (3). Using Finite State Projection (FSP) [25], the state space can be bounded and the time evolution of probability state vectors is given by

(5) $$P_{i}\left(t_{2}\right)={\left[exp\left(W\left(t_{2}-t_{1}\right)\right)\right]}_{ij}P_{j}\left(t_{1}\right)$$

where, $t_{2},t_{1}$ are final and initial time points, respectively. Thus, Equation (5) yields the probability of being in some state *i* at time $t_{2}$ from an initial condition at time $t_{1}$. These conditional probabilities are used to compute the likelihood of producing the synthetic trajectory as a function of the rate parameters (see Equation (15)). The likelihood is then maximized to determine the optimum values of the parameters, thus inferring details of the model. It is important to note that DM ignores mRNA and dimer dynamics and hence is less detailed than the model (Equation (1)) created to generate the synthetic data. The choice of this reduced model is motivated to lower computational cost while constructing a model that is sufficiently fine-grained compared to CGM and MaxCal, outlined next.

#### 2.1.3. CGM Model for Inference

The coarse-grain model (CGM) uses an approach where mass-action (MA) models are combined with CME [29,30,31,32]. Specifically, there is only one reaction describing the time evolution of protein number *A* given by Equation (6). This is akin to a mass-action approach where coupling with other species to capture feedback is modeled in an indirect manner by assuming an ad hoc functional form for the effective rate *X*. Specifically, *X* is given by a Hill type function invoking a cooperative model with n=2 defined in Equation (6). The parameter $g_{2}$ is the basal rate in the absence of any positive feedback and the second term in *X* monotonically increases with *A* approaching an asymptotic value of $g_{2}^{*}$, successfully capturing auto-activation. In this model, the rate in the activated state is $g_{2}+g_{2}^{*}$ and *K* is a parameter. Protein *A* is degraded with rate $r_{2}$.

(6) $$\begin{array}{ccc} & & \alpha \overset{X}{\to }\alpha +A\;\;;\;\;A\overset{r_{2}}{\to }⊘\\ & & X=g_{2}+g_{2}^{*}\frac{A^{n}}{A^{n}+K} \end{array}$$

While traditional MA models use similar functional forms to describe the time evolution of average protein number, adopting Equation (6) within Chemical Master Equation (CME) framework allows stochastic modeling. Thus, the combined approach of CME and MA (CME + MA) computes the probability ($P(N_{A};t)$) of protein number ($N_{A}$) at a given time *t*. Specifically, the time evolution equation of $P(N_{A};t)$ corresponding to the reaction scheme in Equation (6) is given by

(7) $$\begin{array}{ccc} \frac{dP(N_{A},t)}{dt} & = & W_{1}^{cgm}\left(N_{A}-1\right)P\left(N_{A}-1,t\right)+W_{2}^{cgm}\left(N_{A}+1\right)P\left(N_{A}+1,t\right)\\ & - & \left[W_{1}^{cgm}\left(N_{A}\right)+W_{2}^{cgm}\left(N_{A}\right)\right]P\left(N_{A},t\right) \end{array}$$

where $W_{1}^{cgm}\left(N\right)=g_{2}+g_{2}^{*}\frac{N^{n}}{N^{n}+K}$ and $W_{2}^{cgm}\left(N\right)=r_{2}N$. Using the matrix formulation, similar to DM, the likelihood of input trajectories can be calculated as a function of $g_{2},g_{2}^{*},K,r_{2}$. Maximization of the likelihood infers model parameters $g_{2}$, $g_{2}^{*}$, *K*, and $r_{2}$.

#### 2.1.4. MaxCal Model for Inference

MaxCal modeling of SGAA and other circuits have been described in our earlier work [32,34,35]. Here we give a brief outline of the method for SGAA. Maximum Caliber maximizes the path entropy subject to constraints. We define two random variables, $\ell_{\alpha }$ and $\ell_{A}$, to define microscopic trajectories between a time interval *t* and t+Δt. The probabilities of these paths are denoted as $P_{\ell_{\alpha },\ell_{A}}$. Variable $\ell_{\alpha }$ tracks the production of proteins—in the time interval between *t* and t+Δt—ranging between 0 and *M* (predefined maximum) while $\ell_{A}$ is the number of proteins from the previous step that have not undergone degradation, i.e., $0<\ell_{A}<N_{A}$. The first term in *C* (in Equation (8)) is the path entropy and the remaining three terms denote three constraints on average production, average degradation, and a correlation between protein production and the proteins present. The constraints are imposed by Lagrange multipliers $h_{\alpha }$, $h_{A}$ and $K_{A}$, respectively.

(8) $$\begin{array}{c} C=-\sum_{\ell_{\alpha }=0}^{M}\sum_{\ell_{A}=0}^{N_{A}}P_{\ell_{\alpha },\ell_{A}}\log P_{\ell_{\alpha },\ell_{A}}+h_{\alpha }\sum_{\ell_{\alpha }=0}^{M}\sum_{\ell_{A}=0}^{N_{A}}\ell_{\alpha }P_{\ell_{\alpha },\ell_{A}}+\\ h_{A}\sum_{\ell_{\alpha }=0}^{M}\sum_{\ell_{A}=0}^{N_{A}}\ell_{A}P_{\ell_{\alpha },\ell_{A}}+K_{A}\sum_{\ell_{\alpha }=0}^{M}\sum_{\ell_{A}=0}^{N_{A}}\ell_{\alpha }\ell_{A}P_{\ell_{\alpha },\ell_{A}} \end{array}$$

Maximizing the caliber subject to the three constraints yield the probability of these micro trajectories as

(9) Pℓα,ℓA=Q−1NAℓAexp(hαℓα+hAℓA+KAℓαℓA)Q=∑ℓα=0M∑ℓA=0NANAℓAexp(hαℓα+hAℓA+KAℓαℓA)

### 2.2. Two-Gene Toggle Switch (TS) Circuit

Next we consider a two gene mutually repressing circuit called the Toggle Switch (TS), first designed by Collins and colleagues [1].

#### 2.2.1. Generating Synthetic Data

Synthetic data mimicking experimental time trace was created by using the reaction scheme described in reference [21], detailed below:

(10) $$\begin{array}{ccc} & & \alpha \overset{g_{3}}{\to }\alpha +a\;\;;\;\;a\overset{d_{3}}{\to }⊘\\ & & a\overset{p_{3}}{\to }a+A\;\;;\;\;A\overset{r_{3}}{\to }⊘\\ & & \alpha +A\begin{array}{c} \overset{f_{3}}{\to }\\ \overset{}{\underset{b_{3}}{\leftarrow }} \end{array}\alpha^{*}\;\;;\;\;\alpha^{*}\overset{g_{3}}{\to }\;\alpha^{*}+a\;\;;\;\;\alpha^{*}\overset{g_{3}^{*}}{\to }\;\alpha^{*}+b\\ & & \alpha \overset{g_{3}}{\to }\alpha +b\;\;;\;\;b\overset{d_{3}}{\to }⊘\\ & & b\overset{p_{3}}{\to }b+B\;\;;\;\;B\overset{r_{3}}{\to }⊘\\ & & \alpha +B\begin{array}{c} \overset{f_{3}}{\to }\\ \overset{}{\underset{b_{3}}{\leftarrow }} \end{array}\alpha^{\prime }\;\;;\;\;\alpha^{\prime }\overset{g_{3}^{*}}{\to }\;\alpha^{\prime }+a\;\;;\;\;\alpha^{\prime }\overset{g_{3}}{\to }\;\alpha^{\prime }+b \end{array}$$

where mRNA *a* and *b* are transcribed from gene α (different loci) at rate $g_{3}$, that in turn produce proteins *A* and *B*, respectively, at rate $p_{3}$. mRNAs and proteins are degraded at rate $d_{3}$ and $r_{3}$, respectively. Mutual repression is modeled by binding of either protein (*A* or *B*) to the promoter site of α at rate $f_{3}$, altering the promoter state to different expression levels $\alpha^{*}$ (for *A*) and $\alpha^{\prime }$ (for *B*). In the state $\alpha^{*}$, mRNAs of *B* are produced at a rate $g_{3}^{*}$ much less than $g_{3}$, while state $\alpha^{\prime }$ produces mRNAs of *A* at that same slower rate $g_{3}^{*}$, implementing negative feedback. Unbinding of proteins convert the inactivated promoter states ($\alpha^{*}$, $\alpha^{\prime }$) back to the basal state α. For simplicity and as proof of concept, we assume a symmetric model in *A* and *B*. The rate values ($d_{3}=0.5\;s^{-1}$, $p_{3}=0.02\;s^{-1}$, $f_{3}=3.5\times 10^{-6}\;s^{-1}$, $b_{3}=2.0\times 10^{-5}\;s^{-1}$, $g_{3}=0.5\;s^{-1}$, $g_{3}^{*}=2.5\times 10^{-3}\;s^{-1}$, $r_{3}=1.0\times 10^{-3}\;s^{-1}$) were chosen following our earlier work [35]. These values create biologically relevant switching time scales [5] between two states (state 1: low *A*, high *B*; state 2: high *A*, low *B*) while maintaining typical synthesis and degradation rates [27]. Next, the Gillespie algorithm [22] is used to simulate ten replicates of one hundred noisy trajectories (each seven days long) of protein levels that exhibit bistability and serve as our synthetic data to benchmark models.

#### 2.2.2. Detailed Model

To infer network details, we first consider the detailed model (DM) built on a reaction network similar to Equation (10) but neglecting mRNAs. The exact reaction scheme used in DM for TS is described as

(11) $$\begin{array}{ccc} & & \alpha \overset{g_{4}}{\to }\alpha +A\;\;;\;\;A\overset{r_{4}}{\to }⊘\\ & & \alpha +A\begin{array}{c} \overset{f_{4}}{\to }\\ \overset{}{\underset{b_{4}}{\leftarrow }} \end{array}\alpha^{*}\;\;;\;\;\alpha^{*}\overset{g_{4}}{\to }\;\alpha^{*}+A\;\;;\;\;\alpha^{*}\overset{g_{4}^{*}}{\to }\;\alpha^{*}+B\\ & & \alpha \overset{g_{4}}{\to }\alpha +B\;\;;\;\;B\overset{r_{4}}{\to }⊘\\ & & \alpha +B\begin{array}{c} \overset{f_{4}}{\to }\\ \overset{}{\underset{b_{4}}{\leftarrow }} \end{array}\alpha^{\prime }\;\;;\;\;\alpha^{\prime }\overset{g_{4}^{*}}{\to }\;\alpha^{\prime }+A\;\;;\;\;\alpha^{\prime }\overset{g_{4}}{\to }\;\alpha^{\prime }+B \end{array}$$

where the symbols above and below arrows indicate respective reaction rates that will be inferred from the synthetic trajectory data generated from Equation (10). As before, CME arising from this reaction network architecture can be used to compute the probability $P(N_{A}\left(t+\Delta t\right),N_{B}\left(t+\Delta t\right);N_{A}\left(t\right),N_{B}\left(t\right))$ of observing $N_{A}\left(t+\Delta t\right)$ and $N_{B}\left(t+\Delta t\right)$ number of protein molecules at time t+Δt given there were $N_{A}\left(t\right)$ and $N_{B}\left(t\right)$ number of *A* and *B* protein molecules at time *t*. These probabilities are used to compute the likelihood of a given trajectory (see Equation (17)), which can then be maximized to infer rates for this system.

#### 2.2.3. Coarse Grain Model

The coarse grain model (CGM) for TS does not explicitly model protein-promoter complexation unlike DM. Feedback is modeled by an effective production rate of *A* and *B*, given by *X* and *Y*, respectively, defined as

(12) $$\begin{array}{ccc} & & \alpha \overset{X}{\to }\alpha +A\;\;;\;\;A\overset{r_{5}}{\to }⊘\;\;;\;\;\beta \overset{Y}{\to }\beta +B\;\;;\;\;B\overset{r_{5}}{\to }⊘\\ & & X=g_{5}+g_{5}^{*}\frac{1}{B^{n}+K}\;\;;\;\;Y=g_{5}+g_{5}^{*}\frac{1}{A^{n}+K} \end{array}$$

The specific functional form for *X* ensures that large *B* inhibits production of *A*. Similarly, *Y* ensures that *A* inhibits production of *B*. Proteins *A* and *B* degrade with rate $r_{5}$. As before, the reaction network is used to construct the corresponding CME from which the likelihood is computed as a function of model parameters $g_{5}^{*}$, $g_{5}$, *K*, and $r_{5}$. The cooperativity parameter *n* was assumed to be 2, typically assumed in MA models of TS to produce bistability [27]. The inferred values of these parameters are obtained by maximizing the likelihood.

#### 2.2.4. MaxCal

Two variables, $\ell_{\alpha }$ and $\ell_{A}$, were introduced to model SGAA (in Section 2.1.4) to track production of *A* and number of *A* proteins not degraded in a time interval *t* and t+Δt. The same variables were used for protein *A* in TS. Additionally, variables $\ell_{\beta }$ and $\ell_{B}$ were introduced to track the same but for protein *B*. The micro trajectories between time *t* and t+Δt are labeled by stochastic variables $\ell_{\alpha }$, $\ell_{\beta }$, $\ell_{A}$, and $\ell_{B}$, and the corresponding probability is denoted as $P_{\ell_{\alpha },\ell_{A},\ell_{\beta },\ell_{B}}$. The caliber (*C*) is defined as the path entropy (given by the first term on the right hand side of Equation (13)) along with four constraints. First, the average of $\ell_{\alpha }$ and $\ell_{\beta }$ are imposed as constraints with Lagrange multiplier $h_{\alpha }$ to model protein production. Next, the average of $\ell_{A}$ and $\ell_{B}$ are constrained with Lagrange multiplier $h_{A}$ capturing information about degradation. Third, correlations between production and number of existing proteins (of the same protein type) are imposed by constraining average of $\ell_{\alpha }\ell_{A}$ and $\ell_{\beta }\ell_{B}$ by Lagrange multiplier $K_{A\alpha }$ (fourth term in the right hand side of Equation (13)). This term captures any positive feedback that may not be known about a priori (see reference [35] for more). Finally, the average of $\ell_{\beta }\ell_{A}$ and $\ell_{\alpha }\ell_{B}$ are constrained by Lagrange multiplier $K_{A\beta }$ to model cross-correlation (negative feedback) between *A* and *B*.

(13) $$\begin{array}{cc} C= & -\sum_{\ell_{\alpha }=0}^{M}\sum_{\ell_{A}=0}^{N_{A}}\sum_{\ell_{\beta }=0}^{M}\sum_{\ell_{B}=0}^{N_{B}}\left[P_{\ell_{\alpha },\ell_{A},\ell_{\beta },\ell_{B}}\log P_{\ell_{\alpha },\ell_{A},\ell_{\beta },\ell_{B}}\right.\\ \; & +h_{\alpha }\left(\ell_{\alpha }+\ell_{\beta }\right)P_{\ell_{\alpha },\ell_{A},\ell_{\beta },\ell_{B}}+h_{A}\left(\ell_{A}+\ell_{B}\right)P_{\ell_{\alpha },\ell_{A},\ell_{\beta },\ell_{B}}\\ \; & +\left.K_{A\alpha }\left(\ell_{\alpha }\ell_{A}+\ell_{\beta }\ell_{B}\right)P_{\ell_{\alpha },\ell_{A},\ell_{\beta },\ell_{B}}+K_{A\beta }\left(\ell_{\beta }\ell_{A}+\ell_{\alpha }\ell_{B}\right)P_{\ell_{\alpha },\ell_{A},\ell_{\beta },\ell_{B}}\right], \end{array}$$

Maximizing the caliber subject to these constraints gives the path probability in terms of the Lagrange multipliers as

(14) Pℓα,ℓA,ℓβ,ℓB=Q−1NAℓANBℓBexp[hα(ℓα+ℓβ)+hA(ℓA+ℓB)+KAα(ℓαℓA+ℓβℓB)+KAβ(ℓβℓA+ℓαℓB)];Q=∑ℓα=0M∑ℓA=0NA∑ℓβ=0M∑ℓB=0NBNAℓANBℓBexp[hα(ℓα+ℓβ)+hA(ℓA+ℓB)+KAα(ℓαℓA+ℓβℓB)+KAβ(ℓβℓA+ℓαℓB)].

### 2.3. Calculation of Trajectory Likelihood

Parameters of DM, CGM, and MaxCal models are determined by maximizing the likelihood (L) of the synthetic trajectory—mimicking experimental data—recording fluctuating numbers of protein with time.

#### 2.3.1. Calculation of Trajectory Likelihood for SGAA

For SGAA, the likelihood of the trajectory is calculated as

(15) $$L=\prod_{n=1}^{N}P\left(N_{A}\left(t+m\right);N_{A}\left(t=m\left(n-1\right)\right)\right)=\prod_{\left\{i\to j\right\}}P_{(i\to j),m}^{\omega_{(i\to j),m}}\;\;,$$

where *T* is the total snapshots recorded in experiment or synthetic data, N is T/m rounded to the nearest integer and $\omega_{(i\to j),m}$ is the total number of transition from state *i* to *j* over *m* frames. States are given by number of proteins. Typically *m* is chosen in the same range as the average residence time (in frames) in the high and low state. Multiple frames are combined to avoid any spurious jumps in the number of proteins present in the trajectory, not allowed in the MaxCal formulation. Thus, calculating likelihood for transitions over *m* frames allows MaxCal to select reasonable values for the Lagrange multipliers (see reference [34] for further details). The individual transition probability of going from state *i* to state *j* in one time step in MaxCal is computed as

(16) $$P_{i\to j}=\sum_{\ell_{\alpha }=0}^{M}\sum_{\ell_{A}=0}^{i}\delta \left(\ell_{\alpha }+\ell_{A}-j\right)P_{\ell_{\alpha },\ell_{A}}.$$

The transition probability $P_{(i\to j),m}$ over *m* frames—needed for likelihood calculation—can be obtained by arranging single time step transition in a matrix (with i,j as matrix elements) and raising the matrix to the $m^{\mathrm{th}}$ power. FSP formalism—originally devised by Munsky and colleagues [25]—has been used to introduce a sink state and carry out this calculation. Supplemental material in reference [36] provides a general description on how to use FSP formalism for MaxCal, including the calculation of transition probabilities for *m* steps. For DM and CGM models, the calculation of $P_{(i\to j),m}$ require matrix exponentiation following Equation (5) with $t_{2}-t_{1}=m\Delta t$.

#### 2.3.2. Calculation of Trajectory Likelihood for TS

For TS, the likelihood of the trajectory is calculated as

(17) $$\begin{array}{c} L=\prod_{n=1}^{N}P\left(N_{A}\left(t+m\right),N_{B}\left(t+m\right);N_{A}\left(t=m\left(n-1\right)\right),N_{B}\left(t=m\left(n-1\right)\right)\right)\\ =\prod_{\left\{i\to j,k\to l\right\}}P_{(i\to j),(k\to l),m}^{\omega_{(i\to j),(k\to l),m}}\;\;, \end{array}$$

where *T* is total time frames noted in data, N is T/m rounded to the nearest integer, $\omega_{(i\to j),(k\to l),m}$ is the total number of simultaneous transitions from *i* to *j* states (in number of protein A) and *k* to *l* (in number of protein B) over *m* frames, and $N_{A}\left(t\right)$, $N_{B}\left(t\right)$ denote the number of *A*, *B* proteins at time *t* (corresponding to frame m(n−1) where *n* is an integer). The most likely values of $h_{\alpha }$, $h_{A}$, $K_{A\alpha }$, $K_{A\beta }$, and *M* are determined by maximizing the likelihood. As before, transitions between *m* frames were used to avoid large spurious jumps in protein number in one step in the raw data that cannot be easily modeled in MaxCal. The transition probabilities P(j,l,t+1;i,k,t) between two consecutive frames in MaxCal is defined as,

(18) $$P\left(j,l,t+1;i,k,t\right)=P_{i\to j,k\to l}=\sum_{\ell_{\alpha }=0}^{M}\sum_{\ell_{A}=0}^{i}\sum_{\ell_{\beta }=0}^{M}\sum_{\ell_{B}=0}^{k}\delta \left(\ell_{\alpha }+\ell_{A}-j\right)\delta \left(\ell_{\beta }+\ell_{B}-l\right)P_{\ell_{\alpha },\ell_{A},\ell_{\beta },\ell_{B}}.$$

with $P_{\ell_{\alpha },\ell_{A},\ell_{\beta },\ell_{B}}$ defined in Equation (14). The transition probability $P_{(i\to j),(k\to l),m}$ between *m* frames is then calculated by raising the two frame transition probability matrix to the $m^{\mathrm{th}}$ power, similar to the procedure described for SGAA. Similar to SGAA, calculation of the transition probability $P_{(i\to j),(k\to l),m}$ within DM and CGM formalism require matrix exponentiation in contrast to matrix multiplication needed for MaxCal.

## 3. Results

### 3.1. Comparison of Three Models for SGAA

Quantitative comparison of three SGAA models was carried out in terms of their accuracy and efficiency. Accuracy is determined by comparing inferred values of three observables ($p_{\mathrm{eff}},p_{\mathrm{eff}}^{*},r_{\mathrm{eff}}$) against the gold standard used to generate the synthetic data (see Table 1). The true basal production rate $p_{\mathrm{eff}}$ and activated production rate $p_{\mathrm{eff}}^{*}$ are obtained from the parameters in reaction 1 by $p_{\mathrm{eff}}=p\left(g/d\right)$, $p_{\mathrm{eff}}^{*}=p\left(g^{*}/d\right)$. The degradation rate remains the same, $r_{\mathrm{eff}}=r$. For DM, these rates can be extracted as $p_{\mathrm{eff}}=g_{1}$, $p_{\mathrm{eff}}^{*}=g_{1}^{*}$, $r_{\mathrm{eff}}=r_{1}$, where $g_{1}$, $g_{1}^{*}$, and $r_{1}$ are defined in reaction 2. The effective rates for CGM (described by Equation (6)) are calculated as $p_{\mathrm{eff}}=g_{2}$, $p_{\mathrm{eff}}^{*}=g_{2}+g_{2}^{*}$, and $r_{\mathrm{eff}}=r_{2}$. The effective rates using MaxCal are calculated using

(19) $$\begin{array}{ccc} & p_{\mathrm{eff}}\approx \frac{{\langle \ell_{\alpha }\rangle }_{N_{L}}}{\Delta t}, & \;p_{\mathrm{eff}}^{*}\approx \frac{{\langle \ell_{\alpha }\rangle }_{N_{H}}}{\Delta t}\;,\\ & r\left(N\right)=\frac{N-{\langle \ell_{A}\rangle }_{N}}{N\Delta t}, & r_{\mathrm{eff}}\approx \sum_{N}P_{eq}\left(N\right)r\left(N\right), \end{array}$$

where ${\langle \cdots \rangle }_{i}$ denotes the average of an observable for a given $N_{A}=i$, $N_{L}$ is the peak position of the protein number distribution in the low state, $N_{H}$ is the peak in the high state, and $P_{eq}\left(N\right)$ is the probability distribution of *N* proteins at relative equilibrium calculated using FSP [25] (see reference [35] for details). Comparing reported values in Table 1, we conclude that both DM and MaxCal models infer these underlying details reasonably well. CGM however infers a value of $p_{\mathrm{eff}}^{*}$ almost twice the true value.

Next, we provide a comparison of the computational efficiency between the three models by tracking the typical time needed to complete the basic unit of operation invoked in the calculation of the likelihood function. For a given model, we measured the time taken for each likelihood calculation during the entire process of inference. The averages and standard deviations of these times are noted in column 4 in Table 2. For MaxCal, the basic operation is raising the transition matrix to the $m^{\mathrm{th}}$ power, in contrast to the matrix exponentiation required for DM and CGM (see Equation (5)). We note that both CGM and MaxCal have identical matrix dimensions (column 3 in Table 2), constrained by the maximum number of proteins allowed ($N_{max}=92$ reported in column 2 of Table 2). The maximum number of proteins used for FSP calculation was chosen to be significantly higher than the maximum protein number seen in the input trajectory. The total state space dimension is equal to $N_{max}+1$ with the additional state (or “sink” state) accounting for all states with protein numbers greater than $N_{max}$. Despite identical matrix size, a typical step in CGM is slower than MaxCal because matrix exponentiation is slower than matrix multiplication performed *m* times in succession. We notice basic step calculation in DM is significantly slower than MaxCal. This is primarily due to two reasons. First, DM has almost four times larger of a matrix size—compared to MaxCal—due to explicit consideration of the promoter state (basal and activated) in combination with different protein numbers defining the state space. Next, DM requires matrix exponentiation, a much more computationally expensive operation compared to matrix multiplication. We conclude that CGM is less accurate than MaxCal and MaxCal is the method of choice over DM given its efficiency. However, for SGAA, the true power of MaxCal is not reflected since the basic step/operation is in milliseconds for DM and the cumulative time needed for inference (fifth column) is in seconds, feasible on traditional hardware. Nevertheless, the comparison conceptually shows MaxCal’s ability to balance efficiency and accuracy for SGAA, in conjunction with the bigger circuit that we discuss next.

### 3.2. Comparison of Three Models in TS

Next, we provide a quantitative comparison of three models for TS. As before, accuracy is determined by comparing inferred values of the three effective rates, $p_{\mathrm{eff}}$, $p_{\mathrm{eff}}^{*}$, and $r_{\mathrm{eff}}$ against the gold standard used to generate the synthetic data (see Table 3). The true basal production rate $p_{\mathrm{eff}}$, repressed production rate $p_{\mathrm{eff}}^{*}$ are obtained from the parameters in reaction (10) defined as $p_{\mathrm{eff}}=p_{3}\left(g_{3}/d_{3}\right)$ and $p_{\mathrm{eff}}^{*}=p_{3}\left(g_{3}^{*}/d_{3}\right)$. The degradation rate remains the same, $r_{\mathrm{eff}}=r_{3}$. Within the framework of DM, these rates are extracted as $p_{\mathrm{eff}}=g_{4}$, $p_{\mathrm{eff}}^{*}=g_{4}^{*}$, and $r_{\mathrm{eff}}=r_{4}$, where $g_{4}$, $g_{4}^{*}$, and $r_{4}$ are defined in reaction (11). The effective rates for CGM (described by Equation (12)) are calculated as $p_{\mathrm{eff}}=g_{5}+g_{5}^{*}/K$, $p_{\mathrm{eff}}^{*}=g_{5}$, and $r_{\mathrm{eff}}=r_{5}$. The effective rates from MaxCal are calculated as

(20) $$\begin{array}{cc} & p_{\mathrm{eff}}\approx \frac{{\langle \ell_{\alpha }\rangle }_{N_{H},N_{L}}}{\Delta t}=\frac{{\langle \ell_{\beta }\rangle }_{N_{L},N_{H}}}{\Delta t},\\ & p_{\mathrm{eff}}^{*}\approx \frac{{\langle \ell_{\alpha }\rangle }_{N_{L},N_{H}}}{\Delta t}=\frac{{\langle \ell_{\beta }\rangle }_{N_{H},N_{L}}}{\Delta t},\\ & r_{A}\left(N_{A},N_{B}\right)=\frac{N_{A}-{\langle \ell_{A}\rangle }_{N_{A},N_{B}}}{N_{A}\Delta t},\;r_{B}\left(N_{A},N_{B}\right)=\frac{N_{B}-{\langle \ell_{B}\rangle }_{N_{A},N_{B}}}{N_{B}\Delta t},\\ & r_{\mathrm{eff}}\approx \sum_{N_{A}=0}^{\infty }\sum_{N_{B}=0}^{\infty }P_{eq}\left(N_{A},N_{B}\right)r_{A}\left(N_{A},N_{B}\right)=\sum_{N_{A}=0}^{\infty }\sum_{N_{B}=0}^{\infty }P_{eq}\left(N_{A},N_{B}\right)r_{B}\left(N_{A},N_{B}\right), \end{array}$$

where ${\langle \cdots \rangle }_{i,j}$ is the average of a quantity of interest given that there are *i* and *j* number of proteins initially present of type *A* and *B*, respectively, $N_{H}$ and $N_{L}$ are the peaks of the protein number distribution in the basal (unrepressed) and repressed state, respectively, and $P_{eq}\left(i,j\right)$ is the relative equilibrium probability (calculated using FSP [25]) of having $N_{A}=i$ and $N_{B}=j$ proteins. The details of these definitions and results can be found in our earlier work [35]. Comparing the extracted rates against the gold standard, we find DM and MaxCal reliably infer effective rates, similar to their performance with SGAA. CGM however infers rates that differ from the “True” rates by almost an order of magnitude or even more ($p_{\mathrm{eff}}^{*}$ for example).

Next, we provide a comprehensive comparison of the three models in terms of their computational efficiency (see Table 4). Similar to SGAA, DM is significantly slower than MaxCal and CGM primarily due to large matrix size. We have chosen $N_{A,max}=N_{B,max}=59$ resulting in 59×59×2×2+1 = 13,925 states, considering the two states of the promoter and the sink state included in the state space of DM. The enormous dimensional explosion in DM compounded with matrix exponentiation significantly slows down the basic operation to minutes. The estimates of basic steps are obtained using a GPU platform for feasibility reasons. CPU-based computing—used in SGAA–was abandoned as it would have taken an unreasonably long time to complete DM inference. We modified the existing SciPy libraries for calculating the matrix exponential term (needed for basic operation in DM and CGM) using CuPy. To calculate matrix power needed for MaxCal, we used existing CuPy libraries. The timing of the basic step was evaluated on two Nvidia Tesla P100 cards using CUDA version 8.0.

CGM and MaxCal are however much faster than DM. This is primarily because of significantly smaller variable space that CGM and MaxCal uses. Furthermore, we notice MaxCal is even faster compared to CGM due to MaxCal’s reliance on matrix multiplication in contrast to matrix exponentiation. The differences in basic operation (column four in Table 4) translate to marked differences in the total time taken for the entire process of likelihood maximization (reported in column five in Table 4) to infer model parameters. Combining these findings we conclude for TS—similar to SGAA—MaxCal is the preferred method of inference due to its efficiency and ability to reliably infer underlying model parameters. For TS, unlike SGAA, the gain from MaxCal is readily appreciated when using standard GPU hardware with only few nodes.

The example of TS considered above illustrates the typical challenge of inferring network details using detailed models (DM) where multiple genes and species are involved. Genetic circuits larger than TS, i.e., involving more than two expressed proteins, will face even more combinatorial challenges when using models like DM to account for proteins and their promoters. This combinatorial explosion of the state space combined with the need of matrix exponentiation will render detailed models unrealistic for the purposes of inference. Although this challenge can be somewhat mitigated in CGM reducing the state space, CGMs too will face computational challenge, due to their reliance on matrix exponential that will tend to be slower for larger state space (due to multiple genes). Alternate approaches similar to MaxCal should be used where basic operations in likelihood calculation are less burdensome keeping overall computational cost manageable.

## 4. Discussion

Quantitative determination of parameters in a gene network is critical to designing new circuits for synthetic biology applications. However, determining these parameters is challenging due to limited information available on few expressed proteins, much less than the actual number of species involved in such feedback networks. A powerful approach is to mine information-rich stochastic trajectories of protein numbers to infer network details. Three primary modeling schemes were considered to harness information from these noisy trajectories for two specific gene networks: Single Gene Auto Activation (SGAA) circuit and Toggle Switch (TS). The first inferential approach used a detailed modeling (DM) scheme where proteins and their interaction with corresponding genes were explicitly modeled. The second approach employed a coarse grain model (CGM) where Hill type functional forms were invoked to describe feedback, circumventing the need to explicitly model promoter and protein-promoter complexes. The third scheme used the principle of Maximum Caliber (MaxCal) which relies on the maximization of path entropy subject to constraints of protein production, degradation, and feedback. MaxCal—similar to CGM—also avoids explicit consideration of additional molecular species and provides a stochastic description of protein expression trajectories, suitable to analyze noisy gene expression data typically measured in experiments. Using synthetic data generated from a known model, we show that DM accurately infers network details. However, DM is computationally challenging for networks with multiple proteins, even as few as two. The prohibitive computational cost of DM is due to its large state space, resulting in the exponentiation of high dimensional matrices. CGM operates on a lower dimensional state space and hence is more efficient than DM, but still suffers from computational cost for larger circuits due to matrix exponentiation. Moreover, minimal CGMs are less accurate in inferring underlying model details. MaxCal offers the much needed framework both in terms of accuracy and feasibility. In addition, MaxCal provides a systematic way to improve and select models with the same variables but including different correlations as additional constraints. However, adding more constraints will increase the number of parameters (Lagrange multipliers) over which the likelihood function needs to be maximized, slowing down the overall inference process. Nevertheless, MaxCal enjoys the computational efficiency of the basic step—to be iterated multiple times during likelihood maximization—due to relatively smaller matrix size and matrix multiplication operation.

The ability to systematically improve models by adding higher order correlation—due to MaxCal’s inherent top-down nature [32,37]—is another advantage of MaxCal. In contrast, traditional approaches are bottom-up and require first imposing a reaction diagram (such as in DM) or a mechanism (functional forms in CGM). Even with small changes, different reaction networks or functional forms need to be incorporated into the model, restricting systematic model building in a perturbative manner. Furthermore, MaxCal’s reliance on production and degradation variables separately allows the calculation of an effective feedback parameter useful for network characterization, design, and evolution [35,36]. These advantages strongly favor adoption of MaxCal to infer parameters in gene networks from noisy time series protein expression data. However, MaxCal’s performance depends on the availability of basic information. For example, MaxCal model built on only two genes cannot produce oscillation seen in three gene repressilator circuit. MaxCal requires minimal knowledge about the existence of all three genes to correctly model repressilator [36]. Another common challenge with inference is experiments typically measure fluorescence and not protein numbers. Furthermore, fluorescence per protein is not fixed, but random. Direct application of MaxCal on raw experimental data requires experimentally determining the distribution of fluorescence per protein. In the absence of such information, we created synthetic data mimicking noisy fluorescence trajectories and demonstrated MaxCal’s ability to decouple fluorescence noise from gene expression noise and infer underlying circuit details [34,35,36].

It is important to note, MaxCal—in spite of its relative efficiency over DM and CGM— will face increased computational challenge with circuits having multiple different genes due to increased dimensionality of matrices. With the emergence of new mass-spectrometry tools measuring multiple proteins, we are likely to face such data deluge requiring sophisticated tools of inference. Building efficient and reliable models like MaxCal and adoption of GPU platform for additional computational acceleration, as done here, are necessary steps to this direction.

## Figures and Tables

**Table 1:** Comparison of accuracy between three models for SGAA. The first row reports the values of three known (“True”) rates for effective production ($p_{\mathrm{eff}}$) in the basal state, production ($p_{\mathrm{eff}}^{*}$) in the activated state, and protein degradation ($r_{\mathrm{eff}}$) used to generate the synthetic data. The inferred rates using three models, DM (second row), CGM (third row), and MaxCal (fourth row), are compared against each other and the “True” rates indicating that CGM is less accurate than DM and MaxCal. Error bars for rates were obtained by using inference on ten replicates of the input trajectory data.

Method	$p_{\mathrm{eff}}$ (s${}^{-1}$)	$p_{\mathrm{eff}}^{*}$ (s${}^{-1}$)	$r_{\mathrm{eff}}$ (s${}^{-1}$)
True	$5.0\times 10^{-3}$	$50\times 10^{-3}$	$1.0\times 10^{-3}$
DM	$\left(4.2\pm 0.5\right)\times 10^{-3}$	$\left(42\pm 5\right)\times 10^{-3}$	$\left(0.8\pm 0.1\right)\times 10^{-3}$
CGM	$\left(4.1\pm 0.4\right)\times 10^{-3}$	$\left(91\pm 8.9\right)\times 10^{-3}$	$\left(1.3\pm 0.1\right)\times 10^{-3}$
MaxCal	$\left(5.6\pm 0.2\right)\times 10^{-3}$	$\left(43\pm 2.1\right)\times 10^{-3}$	$\left(0.96\pm 0.1\right)\times 10^{-3}$

**Table 2:** Comparison of efficiency between three models for SGAA. Second column reports the maximum number of proteins used in FSP, third column shows the overall matrix dimension, fourth column reports the average time taken (using a CPU platform) for the basic matrix operation needed for a likelihood calculation, and fifth column reports the total time taken during the entire process of likelihood maximization to infer model parameters for the three different models (noted in the first column).

Method	Max *N*	Matrix Size	Unit Operation Time (ms)	Total Time (ms)
DM	92	185×185	10±0.4	1263±69
CGM	92	93×93	3.0±0.2	813±190
MaxCal	92	93×93	0.6±0.5	47±13

**Table 3:** Comparison of accuracy between three models for TS. The first row reports the values of three known (“True”) rates for effective production ($p_{\mathrm{eff}}$) in the basal state, production ($p_{\mathrm{eff}}^{*}$) in the repressed state, and protein degradation ($r_{\mathrm{eff}}$) used to generate the synthetic data. The inferred rates using the three models, DM (second row), CGM (third row), and MaxCal (fourth row), are compared against each other and the “True” rates indicate CGM is less accurate than DM and MaxCal. Error bars for rates were obtained by using inference on ten replicates of the input trajectory data.

Method	$p_{\mathrm{eff}}$ (s${}^{-1}$)	$p_{\mathrm{eff}}^{*}$ (s${}^{-1}$)	$r_{\mathrm{eff}}$ (s${}^{-1}$)
True	$20\times 10^{-3}$	$0.1\times 10^{-3}$	$1.0\times 10^{-3}$
DM	$20.7\times 10^{-3}\pm 5.2\times 10^{-5}$	$0.1\times 10^{-3}\pm 2.6\times 10^{-5}$	$1.0\times 10^{-3}\pm 0.2\times 10^{-3}$
CGM	$170\times 10^{-3}\pm 30\times 10^{-3}$	$2.3\times 10^{-7}\pm 0.7\times 10^{-7}$	$7.0\times 10^{-3}\pm 2.0\times 10^{-3}$
MaxCal	$14.6\times 10^{-3}\pm 0.9\times 10^{-3}$	$0.16\times 10^{-3}\pm 0.02\times 10^{-3}$	$0.7\times 10^{-3}\pm 3\times 10^{-5}$

**Table 4:** Comparison of efficiency between three models for TS. Second column reports the maximum number of proteins used in FSP, third column shows the overall matrix dimension, and fourth column reports the average time taken (using GPU platform) for the basic matrix operation needed for a likelihood calculation and fifth column reports the average of total time taken during the entire process of likelihood maximization to infer model parameters for the three different models (noted in the first column).

Method	Max *N*	Matrix Size	Unit Operation Time (s)	Total Time (s)
DM	59	13,925 × 13,925	223±110	106,878 ± 19,765
CGM	59	3482×3482	5.8±0.4	14,124 ± 12,493
MaxCal	59	3482×3482	0.13±0.01	851±60

## Data Availability

The codes for SGAA and TS are available in Github with links: https://github.com/MaxCalLab/SelfPromo and https://github.com/MaxCalLab/ToggleSwitch, respectively (accessed on 16 March 2021).

## Abbreviations

The following abbreviations are used in this manuscript:

MaxCal	Maximum Caliber
DM	Detailed Model
CGM	Coarse Grain Model
SGAA	Single Gene Auto Activation
TS	Toggle Switch
CME	Chemical Master Equation
FSP	Finite State Projection
